# Vegetation Changes in the Permafrost Regions of the Qinghai-Tibetan Plateau from 1982-2012: Different Responses Related to Geographical Locations and Vegetation Types in High-Altitude Areas

**DOI:** 10.1371/journal.pone.0169732

**Published:** 2017-01-09

**Authors:** Zhiwei Wang, Qian Wang, Xiaodong Wu, Lin Zhao, Guangyang Yue, Zhuotong Nan, Puchang Wang, Shuhua Yi, Defu Zou, Yu Qin, Tonghua Wu, Jianzong Shi

**Affiliations:** 1 Guizhou Institute of Prataculture, Guizhou Academy of Agricultural Sciences, Guiyang, China; 2 Cryosphere Research Station on Qinghai-Xizang Plateau, State Key Laboratory of Cryosphere Science, Northwest Institute of Eco-Environment and Resources, Chinese Academy of Sciences, Lanzhou, China; 3 Graduate University of Chinese Academy of Sciences, Beijing, China; 4 Nanjing Normal University, Nanjing, China; University of Vigo, SPAIN

## Abstract

The Qinghai-Tibetan Plateau (QTP) contains the largest permafrost area in a high-altitude region in the world, and the unique hydrothermal environments of the active layers in this region have an important impact on vegetation growth. Geographical locations present different climatic conditions, and in combination with the permafrost environments, these conditions comprehensively affect the local vegetation activity. Therefore, the responses of vegetation to climate change in the permafrost region of the QTP may be varied differently by geographical location and vegetation condition. In this study, using the latest Global Inventory Modeling and Mapping Studies (GIMMS) Normalized Difference Vegetation Index (NDVI) product based on turning points (TPs), which were calculated using a piecewise linear model, 9 areas within the permafrost region of the QTP were selected to investigate the effect of geographical location and vegetation type on vegetation growth from 1982 to 2012. The following 4 vegetation types were observed in the 9 selected study areas: alpine swamp meadow, alpine meadow, alpine steppe and alpine desert. The research results show that, in these study areas, TPs mainly appeared in 2000 and 2001, and almost 55.1% and 35.0% of the TPs were located in 2000 and 2001. The global standardized precipitation evapotranspiration index (SPEI) and 7 meteorological variables were selected to analyze their correlations with NDVI. We found that the main correlative variables to vegetation productivity in study areas from 1982 to 2012 were precipitation, surface downward long-wave radiation and temperature. Furthermore, NDVI changes exhibited by different vegetation types within the same study area followed similar trends. The results show that regional effects rather than vegetation type had a larger impact on changes in vegetation growth in the permafrost regions of the QTP, indicating that climatic factors had a larger impact in the permafrost regions than the environmental factors (including permafrost) related to the underlying surface conditions.

## Introduction

Vegetation is an important component of terrestrial ecosystems [1] and plays a key regulatory role in energy budgeting and water cycle processes via albedo, roughness, respiration and transpiration at the soil surface [2]. With the development of remote sensing technologies, Normalized Difference Vegetation Index (NDVI) datasets have been widely applied in numerous studies [3–5] as an effective tool for monitoring vegetation growth [6].

Current research [2,7,8] shows that there is a significant regional difference in vegetation changes, and that NDVI exhibits different changing trends at different spatial and temporal scales. For instance, Piao et al. [7] reported that the NDVI in the temperate and cold regions of the Eurasian Continent increased at an annual rate of 0.0005 (*P* = 0.03) from 1982 to 2012. Furthermore, they concluded that there was a turning point (TP) that occurred in 1997, with the NDVI increasing at an annual rate of 0.0018 (*P* < 0.01) before this TP and at an annual rate of -0.0013 (*P* = 0.06) afterwards. In addition, Cheng et al. [2] reported that the trend in Asia-Pacific region was increased at an annual rate of 0.0005 (*P* < 0.01) from 1982 to 2012, and there was also a TP in this region (in 1991), with the NDVI increasing at an annual rate of 0.0026 (*P* < 0.01) before the TP and at a reduced annual rate of 0.0006 (*P* < 0.01) afterwards. At a regional scale, Zhang et al. [8] demonstrated that the NDVI trend increased at an annual rate of 0.0008 (*P* = 0.03) from 1982 to 2011 in the Koshi region in the middle section of the Himalayas, and two TPs occurred in this region (in 1994 and 2000). The trend increased at an annual rate of 0.0019 (*P* = 0.03) before 1994, at an annual rate of -0.0058 (*P* < 0.01) from 1994 to 2000, and at an annual rate of 0.0034 (*P* < 0.01) after 2000.

In addition to regional differences, variations in vegetation type also have an impact on the NDVI. Using the NDVI dataset, Zhang et al. [9] showed that the green-up date of the meadows of the Qinghai-Tibetan Plateau (QTP) occurred earlier than that of the steppes from 1982 to 2011. The product was also applied to investigate the end date of the vegetation growing season, with the results showing that the withering period of the meadows in the QTP occurred later than that of the steppes from 1982 to 2011 [2]. Moreover, an analysis of correlations between the NDVI and sunshine time under different vegetation type conditions (coniferous forest, broad-leaf forest, shrub, meadow, steppe and alpine vegetation) showed that shrub and coniferous forest vegetation was the most significantly correlated to sunshine time [10].

The Qinghai-Tibet Plateau is known as the “third pole of the world", and its vegetation activity is sensitive to climate change [2,9]. In addition, the permafrost regions account for approximately 70% of the total area of the QTP [11], and the permafrost table has a significant water insulating effect [12]. Therefore, permafrost regions have better water conditions and are more favorable to vegetation growth compared with seasonally frozen soil regions [12,13]. The permafrost region of the QTP has unique vegetation types that mainly include alpine steppe (AS), alpine swamp meadow (ASM), alpine meadow (AM) and alpine desert (AD) [9,12,13,14]. These ecosystems have completely different hydrothermal processes as well as different limiting factors for vegetation growth. Therefore, based on hydrothermal conditions, the responses of vegetation in the permafrost regions of the QTP to climate change may be different from those of vegetation in non-permafrost regions. However, permafrost regions are not distinguished from regions with seasonally frozen ground in most current studies [9,15] of the changes in the growth of different vegetation types in the QTP. Instead, the growth conditions or phenological characteristics of vegetation in the permafrost and seasonally frozen soil regions are determined based on the averaged data of such vegetation, which causes difficulties in terms of understanding the changing process of vegetation in permafrost environments. In addition, different geographical locations and vegetation types may also affect the results when determining vegetation changes. Therefore, it is necessary to understand how geological locations and vegetation types affect vegetation growth in the permafrost zone. Hence, in the present study, 9 study area samples, which were distributed uniformly in the permafrost regions of the QTP, were selected. Additionally, the vegetation changes in the corresponding ecosystems of selected areas were analyzed using remote sensing data from 1982 to 2012 to analyze the effects of geological locations and vegetation types about the vegetation growth. The results of the present study could provide a basis for the selection of appropriate spatial scales and vegetation types when using remote sensing data to analyze vegetation growth in the future.

## Materials and Methods

### General situation of the study areas

**Fig 1:**
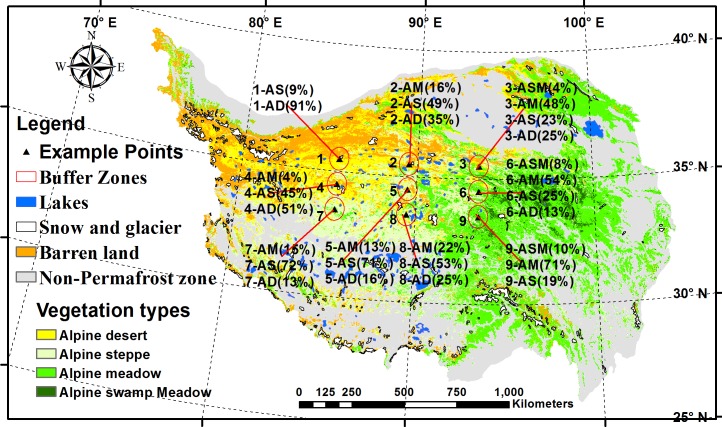
Distribution of the four main vegetation types and 9 study areas (red circle) in the permafrost regions of the QTP. The numbers that precede the “-” represent the codes of study areas (1–9), and the letters that follow the “-” represent the vegetation types (ASM, AM, AS and AD); (%) represents the percentage of area covered by the particular vegetation types in the total study area covered by vegetation.

The permafrost regions of the QTP are located in the high-altitude region of the northern QTP and have an area of approximately 1.5×10^6^ km^2^ (Fig 1). To ensure that the study area samples included more vegetation types at a large spatial scale, 9 study areas were set up in the high-altitude permafrost regions of the Tibet Plateau based on updated vegetation maps of the permafrost zone of the QTP [14]. Excluding the discontinuous area in the permafrost zone of the QTP, a central point (89.5°E, 35.5°N) was first calculated by averaging the values of longitude and latitude. Then, the other 8 points were selected by 4° intervals in the horizontal direction and 1° intervals in the vertical direction to ensure that the study areas included more vegetation types and to avoid locating them in non-permafrost regions. Finally, a buffer area with a radius of 50 km was constructed around the 9 points, as shown in Fig 1.

The field work was conducted in three National Nature Reserves, and the work was conducted under the permission of the Qiangtang National Reserve (study areas 1, 4 and 7), the Aerjin National Nature National Reserve (study areas 2, 5 and 8), and the Kekexili National Nature Reserve (areas 3, 6 and 9). The field studies did not involve endangered or protected species.

### Data source

The datasets used here include the GIMMS NDVI3g dataset, the global standardized precipitation evapotranspiration index (SPEI) dataset and the China Meteorological Forcing Dataset.

The Global Inventory Modeling and Mapping Studies (GIMMS) NDVI dataset used in the present study was acquired by the Advanced Very High Resolution Radiometer (AVHRR) sensor onboard a National Oceanic and Atmospheric Administration (NOAA) satellite. Currently, a third-generation NDVI product has been developed [16], and this product is called the AVHRR GIMMS NDVI3g product, which has a spatial resolution of 1/12°. The GIMMS NDVI3g product is the only dataset that has been continuously updated from the 1980s to the 2010s [2,17]. It is produced every half month, and there are 24 images in each year. The maximum value composites (MVC) method was used for the NDVI product, which can minimize the effects of aerosols and clouds [18]. In addition, the MVC method has been validated using control points in desert areas [7,18,19]. The trends of AVHRR GIMMS NDVI were in overall acceptable agreement with the MODIS NDVI dataset, which were noted in previous researches [20]. Therefore, the 31-year GIMMS NDVI data could provide a robust time series for exploring vegetation change as expressed by NDVI [2].

The global SPEI database [21] offers long-time, robust information about drought conditions at the global scale, with a 0.5° spatial resolution and a monthly time resolution. Currently it covers the period between January 1901 and December 2014.

The China Meteorological Forcing Dataset [22,23] was derived from merging China Meteorological Administration (CMA) station data, Princeton forcing data, GLDAS data, GEWEX-SRB radiation data and TRMM satellite precipitation analysis data by the Institute of Tibetan Plateau Research. It currently covers the period 1972–2015. The spatial resolution and temporal resolution are 0.1° and 3-hr, respectively. The variables include temperature (K), pressure (Pa), specific humidity (kg kg^-1^), wind speed (m s^-1^), downward shortwave radiation (W m^-2^), downward long-wave radiation (W m^-2^) and precipitation rate (mm hr^-1^). Their physical meanings are instantaneous near surface (2 m) air temperature, instantaneous near surface (2 m) air pressure, instantaneous near surface (2 m) air specific humidity, instantaneous near surface (10 m) wind speed, 3-hourly mean (from -1.5 hr to +1.5 hr) surface downward shortwave radiation, 3-hourly mean (from -1.5 hr to +1.5 hr) surface downward longwave radiation and 3-hourly mean (from -3.0 hr to 0.0 hr) precipitation rate respectively. The names of these variables were defined as TEMP, PRES, SHUM, WIND, SRAD, LRAD and PREC, respectively.

### Study methods

The original GIMMS NDVI3g data were rotated by 180°. In this study, these data were converted to TIF files with a WGS-84 projection using the method of geographic correction method with four reference points: top left latitude (90.0-1/24°), top left longitude (-180.0+1/24°), bottom right latitude (-90.0+1/24°) and bottom right longitude (180.0-1/24°). The converted data are shared by the Scientific Data Center for Cold and Arid Regions. The detailed process is reported as follows.

In addition to the NDVI values, the NDVI3g data also include data quality control flag values, which are calculated using the following method:

FLAGVALUES=NDVI3g-floor(NDVI3g/10)*10+1 (1)

NDVI=floor(NDVI3g/10)/1000 (2)

where NDVI3g represents the data value, and floor () represents the downward rounding function. NDVI value was calculated by Eq 2. The FLAG VALUES range from 1 to 7, and the detailed meanings of different flag values are as follows (the information of NDVI and FLAG VALUES can be acquired from https://ecocast.arc.nasa.gov/data/pub/gimms/3g.v0/00READMEgeo.txt): flag values of 1–2 indicate that the pixel value of the point is a good value; flag values of 3–6 indicate that the pixel value of the point is a interpolation value (which is affected by clouds and sensors); and a flag value of 7 indicates that the pixel value of the point is a missing value. In the present study, the NDVI pixel data with flag values of 1 and 2 are selected.

Within the 9 study areas, almost 57 000 points were distinguished as vegetation area at the central position of each pixel of the updated vegetation maps in the permafrost zone of the QTP. They were extracted using geographic information system software to detect the different effects of derived from geographical locations and vegetation types on vegetation changes. NDVI, SPEI and meteorological variables were also extracted at these points.

The growth conditions of the vegetation, SPEI and meteorological variables in the QTP during the 31-year period were analyzed using the least square linear regression method [7,8] and the following equations:

$${\text{y}}_{t}=a+bx_{t}+\varepsilon$$ (3)

$$x_{t}=1,\;2,\;3,\;\ldots ,\;31$$ (4)

where y_t_ represents the time-series of the NDVI, SPEI and meteorological variables; x_t_ represents the year number; a and b represent the parameters of the linear regression equation (a: intercept; b: slope); and ε represents the equation error.

In this study the NDVI, SPEI and meteorological variables were calculated into yearly data using the mean of all data for each year from 1982 to 2012. For the normalization method, the zero-mean normalization (Z-score) was applied to analyze the variables mentioned above. The treated data were in accord with the standard normal distribution. The average and standard deviation of the treated data were 0 and 1 respectively.

Here a piecewise regression model [24], which has been widely applied in many ecological studies, NDVI researches and climate analyses [7,8], was applied to detect potential changes in the NDVI time series. Piecewise regression models are ‘‘broken-stick” models, where two or more lines are joined at unknown points, called “turning points” in previous studies [7,8]. The points could be used as estimates of the thresholds and to determine the width of edge effects [24].

The TPs (turning points) frequently indicated sudden changes in vegetation growth in the analysis of the long-term series NDVI data. They could be calculated using piecewise linear equations, and the following method was used to calculate the TPs [7,8]:

$$y_{t}=\left\{ \begin{array}{l} a_{0}+b_{1}{\text{x}}_{\text{t}}+\varepsilon_{1},{\text{x}}_{\text{t}}\leq \text{j},\text{j}\in \left\{4,5,\ldots ,5,\right\}\\ a_{0}+b_{1}{\text{x}}_{\text{t}}+b_{2}\left({\text{x}}_{\text{t}}-j\right)+\varepsilon_{2},{\text{x}}_{\text{t}}>\text{j} \end{array}\right.$$ (5)

where y_t_ represents the NDVI time-series; x_t_ represents the year number; j represents the year when a TP occurred in the GIMMS NDVI data; a_0_, b_1_ and b_2_ represent the regression coefficients; and ε represents the error. Before a TP, b_1_ is the slope; after a TP, b_1_+b_2_ is the slope. Situations in which there are a scarce number of years before and after the TP are controlled by j ∈ j{4,5,…,5,} [2]. Least-squares linear regression could be selected in estimating j and other coefficients, and a *P* value < 0.05 was considered significant [7].

## Results and Analysis

### NDVI, SPEI and meteorological variables changing patterns and their correlations

**Fig 2:**
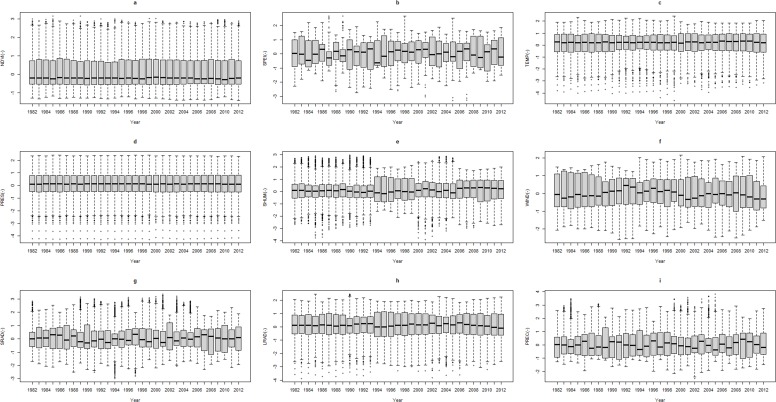
Dynamic of the medians of the NDVI, SPEI and meteorological variables of 9 study areas in the QTP permafrost regions from 1982 to 2012. The boxplot is composed of five types of data: minimum, lower quantile, median, upper quantile and maximum. The dark blank band, the bottom and top of the box, and the top dot and the bottom dot presents the median, lower quantile, upper quantile, minimum and maximum, respectively.

Fig 2 shows the changes in the medians of NDVI, SPEI and meteorological variables of 9 study areas in the QTP permafrost regions. As seen in Fig 2A, there were no significant changes occurring in the vegetation growth conditions over the 31-year time scale (R^2^ = 0.00, *P* = 0.84). However, the statistical significance of the NDVI trend is revealed after assessing the TP in 2000, which is calculated using Eq 5. There was a marginally increased speed (R^2^ = 0.12, *P* = 0.15) before TP and a significantly decreased speed (R^2^ = 0.39, *P* = 0.02) after TP.

As Fig 2B–2I demonstrated, there were significant changes in 5 variables, marginal changes in 1 variable, and no significant changes in 2 variables from 1982 to 2012. The R^2^ and *P* value were 0.01 and 0.60 in SPEI, 0.60 and 0.00 in TEMP, 0.48 and 0.00 in PRES, 0.25 and 0.11 in SHUM, 0.39 and 0.00 in WIND, 0.00 and 0.80 in SRAD, 0.17 and 0.03 in LRAD and 0.59 and 0.00 in PREC respectively, showed in Table 1.

**Table 1:** The R^2^ and *P* values of NDVI, SPEI, TEMP, PRES, SHUM, WIND, SRAD, LRAD and PREC from 1982 to 2012. Temperature, pressure, specific humidity, wind speed, downward shortwave radiation, downward long-wave radiation and precipitation rate were defined as TEMP, PRES, SHUM, WIND, SRAD, LRAD and PREC, respectively.

Variables	R^2^	*P* value
**NDVI**	**0.00**	**0.84**
**SPEI**	**0.01**	**0.60**
**TEMP**	**0.60**	**0.00**
**PRES**	**0.48**	**0.00**
**SHUM**	**0.25**	**0.11**
**WIND**	**0.39**	**0.00**
**SRAD**	**0.00**	**0.80**
**LRAS**	**0.17**	**0.03**
**PREC**	**0.59**	**0.00**

**Fig 3:**
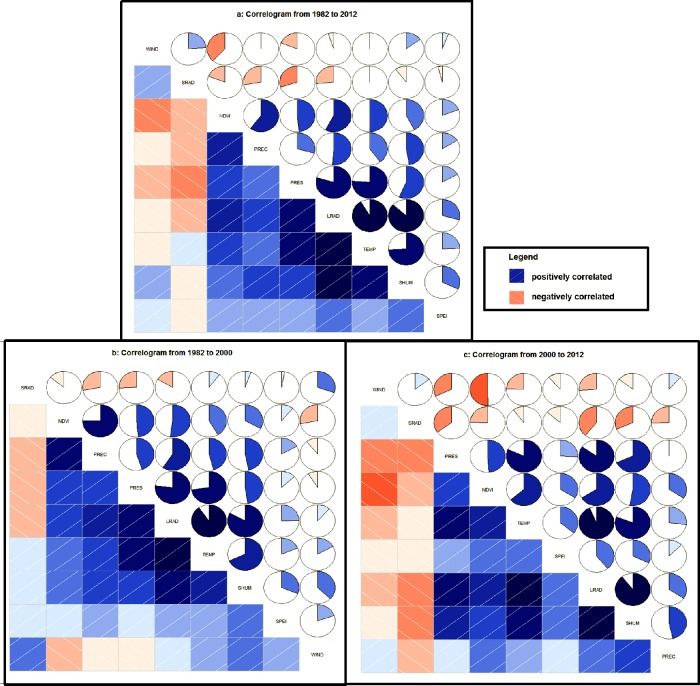
Correlogram of NDVI, SPEI and meteorological variables correlations in different time periods: (a) from 1982 to 2012, (b) from 1982 to 2000 and (c) from 2000 to 2012. The rows and columns of the matrix were reordered by principal component analysis (PCA). Blue box with slashs from the lower left to the upper right indicates that the two crossed variables in the box were positively correlated, and the red box indicates the opposite situation. Deeper colors and higher saturations indicate that the correlation between variables was more significant.

The correlations between these variables are shown in Fig 3. The results demonstrate that the variable, most closely related to NDVI, was PREC from 1982 to 2012. The variable was similar from 1982 to 2000, and only the quantity of the variable was different. However, the variable from 2000 to 2012 was quite different. TEMP was the variable most closely related to NDVI.

### Changes in the NDVI of different vegetation types in different study areas

As shown in Fig 1, a total of 28 vegetation types were observed in the 9 study areas. Relatively abundant vegetation types were observed in the southern permafrost regions of the QTP, with 2 vegetation types in study area 1 and 4 vegetation types in study areas 3 and 6. In the other study area, there were 3 vegetation types.

**Fig 4:**
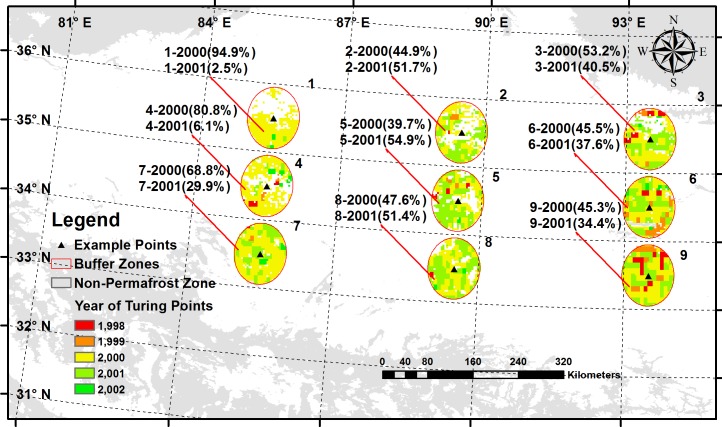
Turning points in each pixel of 9 study areas in the QTP permafrost regions from 1982 to 2012.

Fig 4 demonstrated that there were different TPs at each pixel in 9 study areas. The TPs mainly appeared in 2000 and 2001. Almost 55.1% and 35.0% of the TPs were located in 2000 and 2001. Other TPs in 1998, 1999 and 2002 were 3.5%, 4.3% and 2.1%. Most of the TPs of the study areas were in 2000, and they were in 2001 for study areas 2, 5 and 8. For the quantitatively analyzed regional effects and vegetation type effects on NDVI, the TP was selected as 2000 for each region of the 9 study areas.

From NDVI median, which was calculated using Z-score standardization, there were significant differences in the quantity of NDVI change rate in the 9 study areas, as shown in Table 2. The values of the standardized NDVI median were above 0.0010 in study areas 1–6, and they were below 0.0010 in study areas 7–9. In particular, the values in study areas 1 and 6 were above 0.0020.

**Table 2:** The Z-score standardized NDVI median changed rates of the different vegetation type samples in different time period of different study areas. The letters that precede the “-” represent the vegetation types (ASM, AM, AS and AD), and the letters that follow the “-” represent the codes of the study areas (1–9).

Sample code	Trend from 1982 to 2012	Trend before 2000	Trend after 2000
**AS-1**	0.0034**	**-0.0017**	0.0075*
**AD-1**	0.0039*	-0.0019*	0.0081**
**AM-2**	**0.0018**	**0.0012**	0.0030*
**AS-2**	**0.0014**	0.0017*	**0.0048**
**AD-2**	0.0025**	-0.0007*	0.0066**
**ASM-3**	**-0.0014**	**0.0060**	**-0.0169**
**AM-3**	**-0.0009**	**0.0012**	**0.0039**
**AS-3**	**0.0004**	0.0063*	**-0.0073**
**AD-3**	0.0013*	**0.0015**	0.0055*
**AM-4**	**0.0000**	**0.0007**	**-0.0033**
**AS-4**	0.0013**	0.0013*	0.0012*
**AD-4**	0.0014*	**0.0011**	0.0012*
**AM-5**	**0.00014**	**0.0018**	**0.0012**
**AS-5**	0.0018*	**0.0012**	0.0052*
**AD-5**	0.0012*	**0.0007**	0.0044**
**ASM-6**	0.0021**	0.0018*	0.0025**
**AM-6**	**0.0013**	**0.0039**	0.0015*
**AS-6**	0.0021*	0.0020*	0.0028**
**AD-6**	**-0.0008**	**0.0065**	**-0.0200**
**AM-7**	**0.0004**	**0.0011**	-0.0010*
**AS-7**	**0.0008**	**0.0004**	**-0.0017**
**AD-7**	**0.0008**	**0.0008**	-0.0013*
**AM-8**	**-0.0008**	**-0.0002**	**-0.0006**
**AS-8**	**-0.0009**	**-0.0004**	**-0.0005**
**AD-8**	**0.0005**	**-0.0008**	**0.0005**
**ASM-9**	**0.0008**	**0.0017**	0.0030*
**AM-9**	**0.0003**	**-0.0021**	0.0044**
**AS-9**	**0.0004**	**0.0011**	0.0040*

* Indicates significance level (P < 0.05).

** Indicates significance level (P < 0.01).

## Discussion

### Effect of regional differences on changes in the NDVI

Early research [25] indicated that vegetation in the mid-latitude regions of the northern hemisphere has been greening. Piao et al. [7] found that the NDVI trend in temperate and cold regions of the Eurasian Continent was increased at an annual rate of 0.0005 from 1982 to 2012. Zhang et al. [8] reported that the trend of the Koshi region in the middle section located in the Himalayas had increased at an annual rate of 0.0008 from 1982 to 2011. Chen et al. [2] used the NDVI data from 1982 to 2012 to calculate the vegetation trend in the Asia-Pacific, which increased at an annual rate of 0.0005 during this period. Peng et al. [15] showed that the NDVI trend was increased at an annual rate of 0.0005 from 1982 to 2003, and the NDVI trend of 6 vegetation types were changed at different rates during the same period in the QTP. Compared with the aforementioned results, the NDVI of the permafrost regions in the QTP did not change significantly from 1982 to 2012. The NDVI trend was changed at an annual rate less than 0.0001. From Table 2, the NDVI exhibited different trends in different regions over different time periods relative to a standardized NDVI median.

Based on the calculation using Eq 5, the TP of the permafrost region of the QTP primarily occurred in 2000, which is different from the TPs calculated by Piao et al. [7], Zhang et al. [8] and Chen et al. [2]. Piao et al. [7] pointed that the changes of temperature and precipitation were the main drivers of the change in vegetation productivity in temperate and boreal Eurasia. In this study, SPEI and 7 meteorological variables were selected. The main correlative variables to NDVI were different in different study time period from the correlogram between NDVI and these 8 parameters. The most correlative variables were precipitation and surface downward longwave radiation from 1982 to 2012 and from 1982 to 2000 as Fig 3A and 3B showed. However, these variables were changed into temperature and surface downward longwave radiation from 2000 to 2012 as Fig 3C showed. Therefore the precipitation, surface downward longwave radiation and temperature were the main contributors to vegetation productivity from 1982 to 2012.

A comparative analysis of the standardized NDVI median rates in 9 study areas before and after the TP showed that the slope values of the northern regions were significantly greater than those of the southern regions. Study area 6 was located in the source region of the three rivers, and a significant greening phenomenon occurred in this source region. One of the reasons for this phenomenon could be the implementation of environmental protection policy by local government [26].

The spatial distribution of global NDVI linear trend 2000–2010 seasonally integrated observations [25] shows that the NDVI trend of large vegetation areas in the arctic region exhibited significant increasing trends where permafrost is widely distributed. However, the result [20] pointed that the NDVI trend in the QTP permafrost region, which was located by permafrost, was not significantly increased. The phenomenon may have been caused by differences between the climatic conditions in the permafrost regions of the QTP and Arctic region. The active layers of the permafrost regions of the QTP have relatively large thicknesses and relatively low overall soil water content [27]; therefore, soil water content may be a limiting factor for vegetation growth.

### Effect of differences in vegetation type on NDVI

Previous researches [2,7] have showed that there were differences among growth conditions and vegetation phenophase changes in different vegetation types in the QTP. The present study showed that the NDVI of 4 vegetation types within any one of the 9 study areas in the permafrost regions of the QTP changed with significant level at the same rate from 1982 to 2012 (Table 2). For example, the standardized NDVI median trend were 0.0034 and 0.0039 for alpine steppe and alpine desert in study area 1, 0.0013 and 0.0014 for alpine steppe and alpine desert in study area 4, and 0.0021 and 0.0021 for alpine swamp meadow and alpine desert in study area 6 from 1982 to 2012. The differences of standardized NDVI median trend among different vegetation types in same study area were below 0.0005. However, the differences of standardized NDVI median trend in same vegetation types in different study areas were clear distinct. The values of alpine desert standardized NDVI median trend in study areas 1, 4 and 6 were 0.0034, 0.0013 and 0.0021, respectively.

The vegetation types in the aforementioned studies can be differentiated by the large areas in the eastern region of the QTP classified as meadows, whereas large areas of the western region of the QTP were classified as steppe and small areas such as forests and shrubs were not discussed in this region. The permafrost region of the QTP is extremely sensitive to climate change and has experienced warming and melting trends in recent decades [28]. Degradation of the permafrost of the QTP has been increasingly severe [27,29] in the last decade compared with previous decades and has resulted in the thickening of the active layers, which in turn has caused a series of hydrothermal changes that have an important impact on vegetation changes. The NDVI in study area 6 was increased significantly, which is inconsistent with that of other study areas and previous studies [8]. The reason could be concluded that the permafrost table has a water insulating effect and the thickening of the active layers resulted in more water generated around the upper limit of the permafrost and an increase in the temperature of the soil surface, which is favorable to vegetation growth and development [13]. Moreover, study areas 5, 6 and 9 were located in the source regions of three rivers, where environmental protection and ecological improvement was enhanced after 2000 when the importance of protecting water sources was realized [26]. Thus, the vegetation in this region continued to grow. Beck et al. [30] also found that the growth of vegetation in the Arctic permafrost regions exhibited a significantly increasing trend after 2000. Therefore, different NDVI changed trends of different vegetation types in the QTP may have resulted from spatial differences instead of vegetation-type differences.

## Conclusions

There were significant vegetation changes in the permafrost regions of the QTP before and after 2000, with the mean NDVI increasing at an annual rate of 0.0006 before 2000 and decreasing at an annual rate of 0.0008 after 2000. An investigation of the change trends of the NDVI of the 4 vegetation types in the 9 vegetation survey areas showed that the growth rates in the permafrost regions of the QTP increased differently in the different regions. In addition, the NDVI of different vegetation types within the same study area changed at the same general rate. Therefore, the regional effect had a larger impact than the vegetation-type effect on the growth conditions of the vegetation in the permafrost regions of the QTP.

## Supporting Information

S1 Fig A map of Fig 1 exported from ArcGIS 10.1. (PDF)

S2 Fig A map of Fig 4 exported from ArcGIS 10.1. (PDF)

S1 Dataset NDVI, SPEI and climate data for Figs 2 and 3. (ZIP)

## References

[1] Jong RD, Bruin SD, Wit AD, Schaepman ME, Dent DL. Analysis of monotonic greening and browning trends from global NDVI time-series. Remote Sensing of Environment 2011; 115: 692–702.

[2] Chen BZ, Xu G, Coops NC, Ciais P, Innes JL, Wang GY, et al Changes in vegetation photosynthetic activity trends across the Asia–Pacific region over the last three decades. Remote Sensing of Environment 2014; 144: 28–41.

[3] Bhatt US, Walker DA, Raynolds MK, Bieniek PA, Epstein HE, Comiso JC, et al Recent declines in warming and vegetation greening trends over Pan-Arctic tundra. Remote Sensing 2013; 5: 4229–4254.

[4] Vrieling A, Leeuw JD, Said MY. Length of growing period over Africa: variability and trends from 30 years of NDVI time series. Remote Sensing 2013; 5: 982–1000.

[5] Jiang N, Zhu W, Zheng Z, Chen G, Fan D. A comparative analysis between GIMSS NDVIg and NDVI3g for monitoring vegetation activity change in the northern hemisphere during 1982–2008. Remote Sensing 2013; 5: 4031–4044.

[6] Tucker CJ. Red and photographic infrared linear combinations for monitoring vegetation. Remote Sensing of Environment 1979; 8: 127–150.

[7] Piao SL, Wang XH, Ciais P, Zhu B, Wang TA, Liu JI. Changes in satellite-derived vegetation growth trend in temperate and boreal Eurasia from 1982 to 2006. Global change Biology 2011; 17: 3228–3239.

[8] Zhang YL, Gao JG, Liu LS, Wang ZF, Ding MJ, Yang XC. NDVI-based vegetation changes and their responses to climate change from 1982 to 2011: A case study in the Koshi River Basin in the middle Himalayas. Global and Planetary Change 2013; 108: 139–148.

[9] Zhang GL, Zhang YJ, Dong JW, Xiao XM. Green-up dates in the Tibetan Plateau have continuously advanced from 1982 to 2011. Proceedings of the National Academy of Sciences 2013; 110: 4309–4314. 10.1073/pnas.1210423110 PMC3600495 23440201

[10] Chen ML, Chen BZ, Innes JL, Wang GY, Dou XM, Zhou TM, et al Spatial and temporal variations in the end date of the vegetation growing season throughout the Qinghai–Tibetan Plateau from 1982 to 2011. Agricultural and Forest Meteorology 2014; 189–190: 81–90.

[11] Jin HJ, Li SX, Wang SL, Zhao L. Impacts of climatic change on permafrost and cold regions environments in China. Acta Geographica Sinica 2000; 55:161–173.

[12] Chen SY, Liu WJ, Qin X, Liu YS, Zhang TZ, Chen KL, et al Response characteristics of vegetation and soil environment to permafrost degradation in the upstream regions of the Shule River Basin. Environmental Research Letters 2012; 7: 045406.

[13] Yi SH, Zhou ZY, Ren SL, Xu M, Qin Y, Chen SY, et al Effects of permafrost degradation on alpine grassland in a semi-arid basin on the Qinghai–Tibetan Plateau. Environmental Research Letters 2011; 6: 045403.

[14] Wang ZW, Wang Q, Zhao L, Wu XD, Yue GY, Zou DF, et al Mapping the vegetation distribution of the permafrost zone on the Qinghai-Tibet Plateau. Journal of Mountain Science 2016; 13: 1035–1046.

[15] Peng J, Liu ZH, Liu YH, Wu JS, Han Y. Trend analysis of vegetation dynamics in Qinghai–Tibet Plateau using Hurst Exponent. Ecological Indicators. 2012; 14: 28–39.

[16] Pinzon J, Tucker C. A non-stationary 1981–2012 AVHRR NDVI3g time series. Remote Sensing 2014; 6: 6929–6960.

[17] Beck HE, Mcvicar TR, van Dijk AI, Schellekens J, de Jeu R, Bruijnzeel LA. Global evaluation of four AVHRR–NDVI data sets: intercomparison and assessment against Landsat imagery. Remote Sensing of Environment 2011; 115: 2547–2563.

[18] Tucker CJ, Pinzon JE, Brown ME, Slayback DA, Pak EW. An extended AVHRR 8-km NDVI dataset compatible with MODIS and SPOT vegetation NDVI data. International Journal of Remote Sensing 2005; 26: 4485–4498.

[19] Zeng FW, Collatz GJ, Pinzon JE, Alvaro L. Evaluating and quantifying the climate-driven interannual variability in Global Inventory Modeling and Mapping Studies (GIMMS) Normalized Difference Vegetation Index (NDVI3g) at global scales. Remote Sensing 2013; 5: 3918–3950.

[20] Fensholt R, Proud SR. Evaluation of Earth Observation based global long term vegetation trends-Comparing GIMMS and MODIS global NDVI time series. Remote Sensing of Environment 2012; 119: 131–147.

[21] Beguería S, Vicente-Serrano SM, Reig F, Latorre B. Standardized precipitation evapotranspiration index (SPEI) revisited: parameter fitting, evapotranspiration models, tools, datasets and drought monitoring. International Journal of Climatology 2014; 34: 3001–3023.

[22] Chen YY, Yang K, He J, Qin J, Shi JC, Du JY, et al Improving land surface temperature modeling for dry land of China. Journal of Geophysical Research 2011; 116: D20104.

[23] He J, Yang K. China meteorological forcing dataset. Cold and Arid Regions Science Data Center at Lanzhou 2011.

[24] Toms JD, Lesperance ML. Piecewise regression: a tool for identifying ecological thresholds. Ecology 2003; 84: 2034–2041.

[25] Sobrino JA, Julien Y. Global trends in NDVI-derived parameters obtained from GIMMS data. International Journal of Remote Sensing 2011; 32: 4267–4279.

[26] Liu XF, Zhang JS, Zhu XF, Pan YZ, Liu YX, Zhang DH, et al Spatiotemporal changes in vegetation coverage and its driving factors in the Three-River Headwaters Region during 2000–2011. Journal of Geographical Sciences 2014; 24: 288–302.

[27] Wu QB, Zhang T, Liu Y. Permafrost temperatures and thickness on the Qinghai-Tibet Plateau. Global Planetary Change 2010; 72: 32–38.

[28] Yang M, Nelson FE, Shiklomanov N, Guo D, Wan G. Permafrost degradation and its environmental effects on the Tibetan Plateau: a review of recent research. Earth-Science Reviews 2010; 103: 31–44.

[29] Chen H, Zhu QA, Peng CH, Wu N, Wang YF, Fang XQ, et al The impacts of climate change and human activities on biogeochemical cycles on the Qinghai-Tibetan Plateau. Global Change Biology 2013; 19: 2940–2955. doi: 10.1111/gcb.12277 23744573 10.1111/gcb.12277

[30] Beck PS, Goetz SJ. Corrigendum: Satellite observations of high northern latitude vegetation productivity changes between 1982 and 2008: ecological variability and regional differences. Environmental Research Letters 2012; 7: 029501.

