# Supplementary figures and images for: Vegetation Changes in the Permafrost Regions of the Qinghai-Tibetan Plateau from 1982-2012: Different Responses Related to Geographical Locations and Vegetation Types in High-Altitude Areas
Source: PLoS One. 2017 Jan 9;12(1):e0169732. doi: 10.1371/journal.pone.0169732 (PMC5222499; doi:10.1371/journal.pone.0169732)

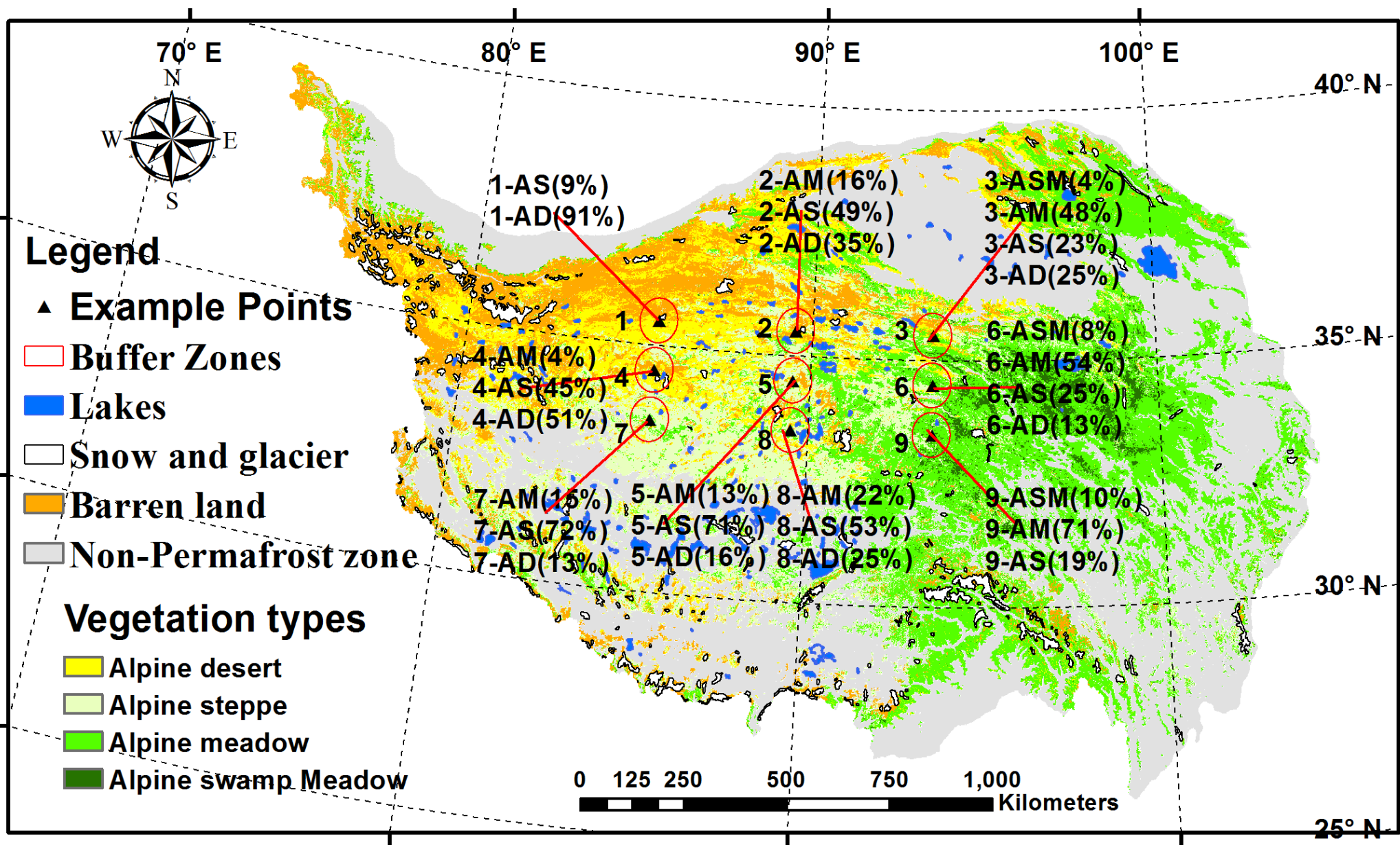

Supplement: S1 Fig — (PDF) [file pone.0169732.s001.pdf]

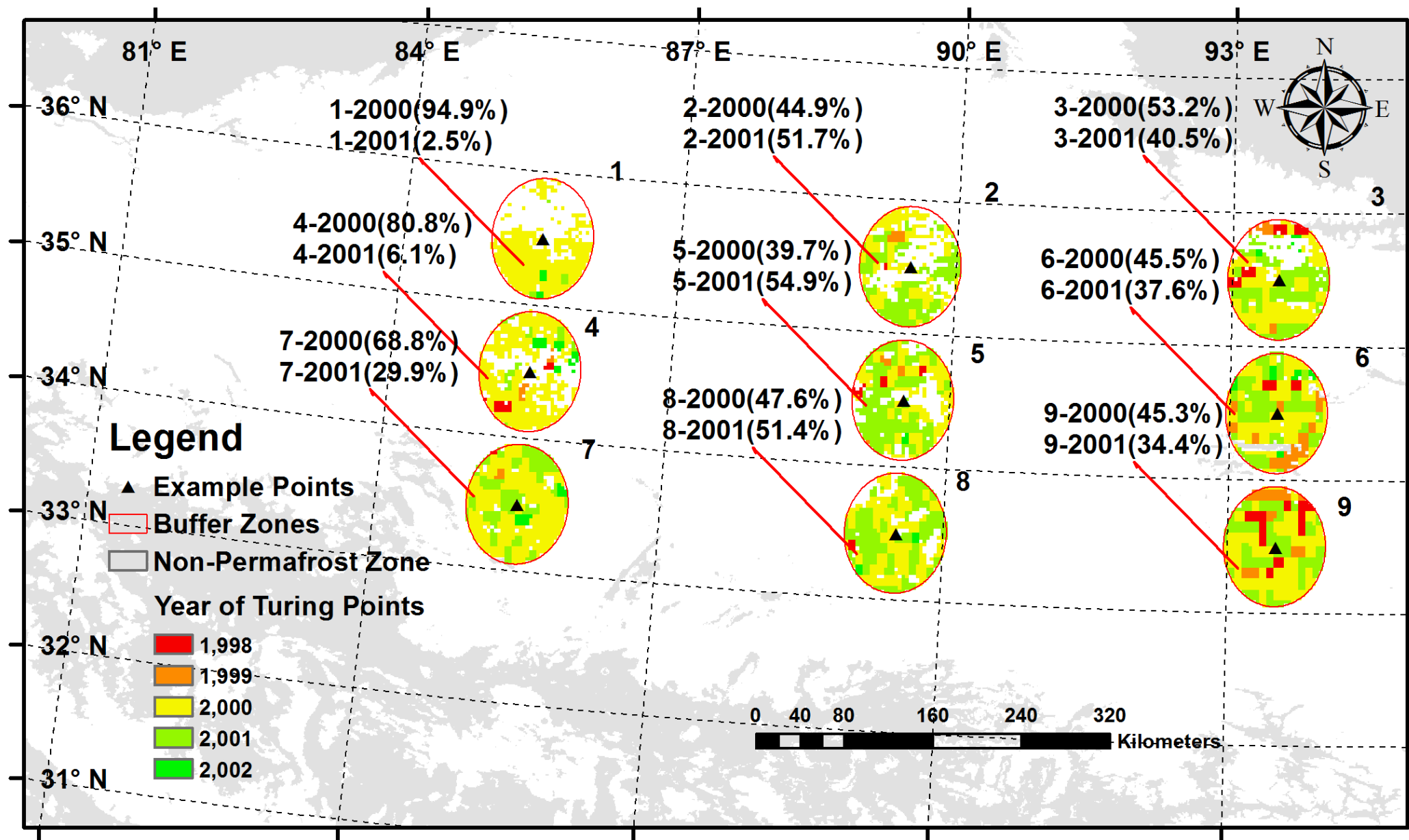

Supplement: S2 Fig — (PDF) [file pone.0169732.s002.pdf]
